# The Amusic Brain: Lost in Music, but Not in Space

**DOI:** 10.1371/journal.pone.0010173

**Published:** 2010-04-21

**Authors:** Barbara Tillmann, Pierre Jolicœur, Masami Ishihara, Nathalie Gosselin, Olivier Bertrand, Yves Rossetti, Isabelle Peretz

**Affiliations:** 1 Université Lyon 1, Lyon, France; 2 CNRS-UMR 5020, Lyon, France; 3 BRAMS Laboratory and Department of Psychology, Université de Montréal, Montréal, Quebec, Canada; 4 Max-Planck-Institute - CBS, Leipzig, Germany; 5 INSERM U821, Lyon, France; 6 INSERM U864; Hospices Civils de Lyon, Lyon, France; University College London, United Kingdom

## Abstract

Congenital amusia is a neurogenetic disorder of music processing that is currently ascribed to a deficit in pitch processing. A recent study challenges this view and claims the disorder might arise as a consequence of a general spatial-processing deficit. Here, we assessed spatial processing abilities in two independent samples of individuals with congenital amusia by using line bisection tasks (Experiment 1) and a mental rotation task (Experiment 2). Both amusics and controls showed the classical spatial effects on bisection performance and on mental rotation performance, and amusics and controls did not differ from each other. These results indicate that the neurocognitive impairment of congenital amusia does not affect the processing of space.

## Introduction

Most humans are born with the potential to speak and to make music. For the majority of individuals who are musically untrained, this fundamental human trait is expressed by music listening, occasional dancing, and occasional singing. The propensity to engage in music ultimately gives rise to a sophisticated music processing system that is acquired largely implicitly by experience. However, a minority of individuals never acquire this core musical system, either in part or at all, despite normal hearing and other cognitive functions and normal exposure to music. This condition concerns 4% of the general population [1] and is termed congenital amusia [2]. This disorder is akin to other developmental disorders, such as congenital prosopagnosia, dyscalculia, dysphasia, and dyslexia.

Congenital amusia is thought to result from a musical pitch-processing disorder. What amusics seem to be lacking are pitch-processing abilities that are normally and incidentally acquired by ordinary listeners early in life, and that are essential for normal music processing. Indeed, amusic individuals are impaired in processing pitch directions [3] and detecting pitch deviations that are smaller than one semitone in tone sequences [4] as well as in tone pairs [2]. Given that amusic individuals are probably born with such an elemental deficit (normal infants' pitch acuity is in the order of half a semitone [5]), they probably have not assimilated the structure of musical scales nor acquired the sophisticated tonal knowledge that normally developing individuals implicitly acquire via mere exposure [6]. Thus, a perceptual system that is unable to perceive small pitch changes is likely to miss an essential part of musical structure [7].

Indeed, amusic individuals fail to recognize a familiar tune without the aid of the lyrics, are unable to detect when they sing out-of-tune, and have severe difficulties to judge if two melodies are the same or different, especially on the pitch dimension. They also show little sensitivity to the presence of obvious pitch violations in melodies and of dissonant chords in classical music [8]. The pitch-processing deficit can even affect the processing of speech intonation [9]. An associated rhythm deficit that is observed in about half of amusics seems to result from pitch variations in melodies [2], [8], [10]. When presented with rhythmic sequences from which pitch variations are removed, amusic individuals discriminate them as well as control participants [11]. In sum, the core deficit in amusia concerns the processing of pitch.

This musical pitch-processing disorder represents a phenotype that serves to identify the associated neuro-genetic factors [12], [13], [14], [15]. However, Douglas and Bilkey [16] have recently challenged this view. They reported that amusics were impaired in a classic mental rotation task and were less influenced by the spatial layout of response keys in a pitch-judgment task, compared to musically-normal participants. This apparent deficit of spatial processing suggested to them an impairment in amusia of a shared mental representation of pitch and space. If confirmed, these results challenge the current search for causal links between musical pitch, brain, and behavior [17]. The goal of the present study was to investigate further this putative spatial deficit in amusia.

The hypothesis of a link between pitch and space is not new. There is evidence that pitch processing interacts with visual space representations in the normal brain (e.g., [18], [19], [20], [21], [22]). As also shown by Douglas and Bilkey [16], spatial associations with pitch can be revealed by stimulus-response compatibility effects [19], [20]. Responses may slow down when the spatial arrangement of response keys conflicts with the spatial descriptors of the to-be-judged materials, such as a spatially lower response key position to respond to a higher pitch (or a spatially higher key position for a lower pitch) relative to spatially-compatible pairings (e.g., higher key position for higher pitch). Similar spatial compatibility effects have been reported for number processing (e.g., right vs. left response keys to respond to larger vs. smaller numbers, or vice-versa [23], [24], [25], [26]). Recent evidence suggests that these spatial representations are specific to pitch and number domains, respectively. Notably, Beecham et al. [27] reported spatial stimulus-response compatibility effects for both number and pitch together, but with independence of these effects.

Within the musical domain, an association between pitch and space has been documented in other contexts than the stimulus-response compatibility arrangement. For example, musical expertise can lead to enhanced visuo-spatial processing [28], [29]. Therefore, the possibility that congenital amusia might represent the low end of a continuum from deficit to excellence in pitch processing and its association to spatial processing, as suggested by Douglas and Bilkey, is plausible and thus worthy of further studies.

Our first aim was to further explore the spatial processing deficit that has been revealed by Douglas and Bilkey. Impaired spatial representations can be caused by a distorted representation (thus decreased accuracy, e.g., due to biases) and/or decreased precision (leading to increased variability). These two aspects of spatial representation can be investigated with the line bisection task [30], [31], which was used in Experiment 1.

**Figure 1:**
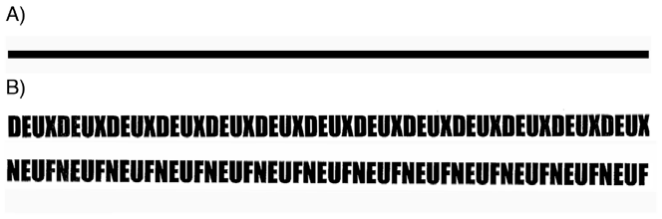
Bisection tasks (Experiment 1). Participants were instructed to mark the midpoint of a straight line as in panel A or a line made of letter strings spelling out small or larger number words (two and nine written in French) as in panel B.

The line bisection task is a widely used tool to assess spatial representations in neglect patients [33], [34] and healthy participants [35]. It mainly allows testing spatial representations for accuracy, notably by measuring the distance between bisection point and veridical midpoint. But it can be also used to assess the precision of spatial representation by measuring inter-trial variability. Previous research has reported pathological cases showing deficits in both accuracy and precision (e.g., hemi-neglect patients [30], [36]) or solely in precision (e.g., [37]). Healthy participants typically bisect the line slightly and systematically to the left of the midpoint, also referred to as pseudo-neglect [38]. In a normal brain, bisection performance is affected by simultaneous number processing, suggesting interactions between mental representations of space and numbers. The influence of numbers on bisection performance is probably mediated by the spatial representation of the mental number line that is arranged from left to right [39], [40]: When lines are made out of number words (see Figure 1), smaller numbers (i.e., two/deux) induce a stronger leftward bias than larger numbers (i.e., nine/neuf).

Experiment 1 used the bisection task as a tool to determine the nature of the putative spatial deficit of amusics, notably by measuring accuracy and precision as well as the modulation of the representations with suspected associations (i.e., numbers). The use of the bisection paradigm was further motivated by a recent data set showing an influence of musical expertise on bisection performance: musicians bisect more accurately, closer to the center, or show a rightward bias [41]. Musicians and amusic individuals represent two extremes of the spectrum of musical ability: while improved spatial abilities have been reported for musicians also with other tasks (e.g., [28], [29]), impaired spatial processing has been reported for amusic individuals [16].

Here, a group of amusics and a group of controls were tested in two bisection conditions, with the straight line (i.e., the classical version) and a number line made out of small or large numbers. Based on Douglas and Bilkey's claim that amusia is “strongly related to a deficit in spatial processing” (p. 913) and the previously observed relations between the processing of space and numbers (e.g., [23], [24], [26]), altered bisection performance was expected for congenital amusics in all conditions in comparison to controls. If, however, spatial representations are distinct for pitch and numbers, as suggested by Beecham et al. [27], congenital amusics may show normal performance with the number lines, despite their pitch deficit.

Contrary to expectations, we observed in Experiment 1 that amusics' bisection performance did not differ from controls' performance neither for accuracy nor precision, thus failing to reveal a deficit in spatial processing. Therefore we decided to revisit the spatial processing capacities of amusics with the mental rotation task [32] used by Douglas and Bilkey [16]. In order to assess a possible negative result, Experiment 2 replicated Douglas and Bilkey but improved the testing conditions (i.e., statistical power and methodology), as described below.

## Results

### Experiment 1: Bisection tasks

Accuracy in bisection was computed in mm-deviation from the midpoint of each line. As shown in Table 1, bisection performance of amusics and controls did not differ. For the lines, both groups bisected left of true midpoint and to the same extent (*p*>.74; two-tailed *t*-test). The leftward bisection differed significantly from the midpoint for both amusics (t(10) = 3.55, p = .006) and controls (t(10) = 4.16, p = .003). For the number lines, the observed leftward bias was modulated by number magnitude and this modulation did not differ between amusics and controls: Bisections of lines made of DEUX (i.e., two) were biased more toward the left than for those made of NEUF (i.e., nine), reflecting the influence of the mental number line. This was supported by an ANOVA with Group (amusics vs. controls) and Condition (small vs. large number lines) considered as between- and within-subjects factors, respectively, which yielded a main effect of Condition (*F*(1, 20)  = 7.84, *p*<.01), but no effect of Group or interaction of Group and Condition (*p*s>.57).

**Table 1:** Bisection tasks (Experiment 1): Bisection performance expressed in cm-deviation from the midpoint obtained in amusics and in controls.

Group	Straight line	Number lines	
		Small	Large
Amusics	−.23 (.07)	−.09 (.04)	−.03 (.05)
Controls	−.26 (.06)	−.14 (.09)	−.09 (.08)

Standard errors are indicated in parentheses.

To investigate whether amusics might be subject to decreased precision of spatial representations and thus to increased variability of midpoint estimates, additional analyses were performed on within-participant variability between trials for data of straight lines and number lines (Table 2). No group differences were observed in either condition (*p*s>.59). This finding shows that amusics do not exhibit less precise mid-point estimations than normals, which suggests they are not subject to a degraded spatial representation in comparison to normal controls. Furthermore, performance on these two bisection tests was unrelated to the musical abilities scores obtained on the full amusia battery and its contour subtest as well as to the pitch performance in the pitch detection task (all *r*s (20) <|.21|).

**Table 2:** Within-participant variability over trials for amusics and controls for bisection tasks on straight lines and number lines (expressed in cm-deviation; Experiment 1).

Group	Straight lines	Number lines	
		Small	Large
Amusics	.24 (.02)	.27 (.03)	.27 (.04)
Controls	.25 (.02)	.27 (.04)	.26 (.02)

Note: Standard errors are in parentheses.

### Experiment 2: Mental rotation task

**Figure 2:**
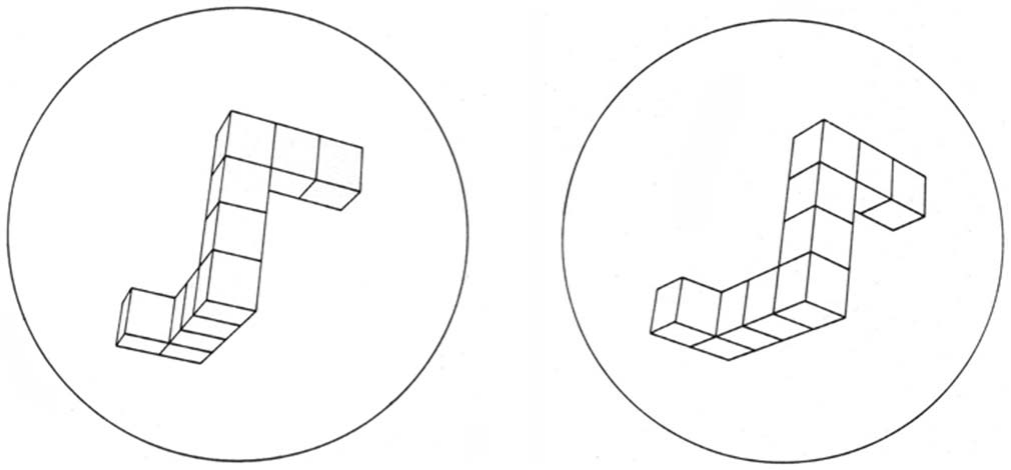
Mental rotation task (Experiment 2). Example of a test pair with two different objects.

Because Experiment 1 failed to find any evidence of spatial deficit in amusia, Experiment 2 examined whether we could replicate amusics' purported difficulties in the mental rotation task [32] (Figure 2). To increase the sensitivity of the experiment and the precision of the measurements in comparison to the study conducted by Douglas and Bilkey [16], we increased here the number of observations from 20 to 160 and we switched from an overall manual test to a computerized version that timed each response.

**Figure 3:**
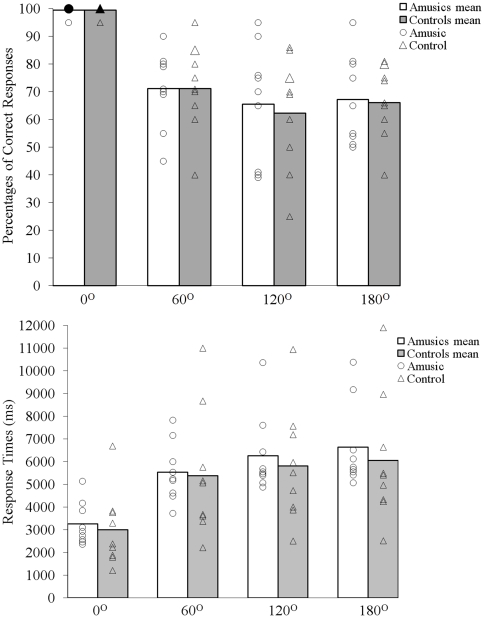
Mental rotation task (Experiment 2). Percentages of correct responses (%, top) and response times (ms, bottom) for the same trials presented as a function of degrees of rotation and groups.

Accuracy (Figure 3 top) and Response Times (RTs; Figure 3 bottom) in mental rotation were analyzed in ANOVAs with Group (amusics or controls) as between-participants factor and Degree of Rotation (0, 60, 120, or 180) as within-participant factor. The most important results were the complete absence of interactions of Group and Degree of Rotation in both the analysis of RTs, *F*(3, 48)  = .212, *p*>.88, and the analysis of accuracy *F*(3, 48)  = .092, *p*>.96). There was no effect of Group either (*p*s>.63). When considering only the training trials (i.e., 24 trials including six items for each rotation, which represented a similar number of trials as in [16]), percentages of correct responses did not differ between the two groups either, t(16)  = −0.318, p = 0.75. However, for all trials, we observed the classical effect of Degree of Rotation on the percentages of correct responses, *F*(3, 48)  = 40.00, *p*<.001, and on the response times, *F*(3, 48)  = 46.84, *p*<.001. To investigate an eventual influence of gender, we ran two additional ANOVAs with Gender as between-participants factor and Degree of Rotation as within-participants factor. For both accuracy and response times, these analyses confirmed the main effect of degree of rotation (*p*s<.0001), but did not reveal any influence of Gender (p*s>.43)*.

Analyses of within-participant variability between items (Table 3) did not reveal any group difference either (*p*s>.73). Finally, as in Experiment 1, performance on the mental rotation task (accuracy, RTs) was unrelated to the musical abilities scores obtained on the full amusia battery and its contour subtest (*r*s (16) *<*|.23|), as well as on the pitch detection task (all *r (12) <*|.16|; four of the matched controls did not participate in the pitch discrimination task).

**Table 3:** For the mental rotation task (Experiment 2): Intra-participant variability over trials for response times *var (ms)* and accuracy *var (%)* presented as a function of degrees of rotation and groups.

		Degrees of rotation			
		0	60	120	180
var (ms)	Amusics	935 (378)	1262 (334)	1156 (316)	1138 (363)
var (ms)	Controls	751 (311)	1170 (364)	1111 (252)	1604 (420)
var (%)	Amusics	.02 (.03)	.44 (.02)	.43 (.03)	.45 (.03)
var (%)	Controls	.02 (.03)	.43 (.03)	.45 (.02)	.47 (.04)

Note: Standard errors are presented in parentheses.

## Discussion

In Experiment 1, we provide evidence that amusics have a bisection performance comparable to that of healthy nonmusician controls, suggesting normal visuo-spatial processing in the amusic brain. Amusic individuals showed normal leftward biases and number biases in bisection performance as previously observed [38], [39], [40]. This result suggests that both visuo-spatial line representations and mental number line representations are preserved in amusia. Both control and amusic participants showed interference between the mental representation of number and space processing (i.e., as reflected here in the bisection performance of number lines), suggesting that this interference is independent of perceivers' pitch representation - whether impaired or unimpaired. Our findings based on the comparison of perceivers with and without pitch deficit are in agreement with Beecham et al.'s [27] observations within (normal) participants, leading them to conclude that different mental spatial representations are involved in number and pitch processing.

A third bisection condition had investigated the influence of simultaneous pitch processing on bisection performance (as in [42]). However, we did not replicate the previously observed pitch influence even for control participants. This failure might be due to our participants being nonmusicians, as suggested by earlier results that had shown spatial pitch effects to be larger in musicians than nonmusicians [19], [20]. The finding suggests that participants in [42] might have had some musical expertise (their musical background had not been reported; see also [43]). Future experiments need to further investigate the influence of pitch presentation and participants' musical expertise on bisection performance in normal populations.

In Experiment 2, using the mental rotation task, we also failed to find any indication of a spatial deficit in amusia. Amusics' performance instead exhibited the typical effect of rotation angle that is found in healthy participants, hence indicating normal construction and spatial transformation of visuo-spatial objects. Thus, the present results do not provide evidence for a spatial deficit in amusia, despite the higher sensitivity of the tests used to evaluate mental rotation performance here as compared to Douglas and Bilkey [16].

In conclusion, with both bisection and mental rotation tasks, we show that amusics' deficit in pitch processing does not co-occur with a deficit in spatial processing. The present findings support the view that congenital amusia is a neurogenetic disorder that affects the processing of pitch selectively. Thus, the disorder remains a rare chance to examine the biological basis of an auditory disorder that is pitch-based by tracing causal links between pitch perception, reduced connectivity in the right fronto-temporal network and genes [44]. In addition to the implications for the neurogenetic origins of congenital amusia, the present results further suggest independent pitch representations and visuo-spatial representations: a pitch deficit does not co-occur with a spatial deficit.

## Materials and Methods

### Experiment 1: Bisection tasks

#### Participants

The amusic group and the control group each comprised 11 adults, who were matched for gender, age, education, and musical training (Table 4). All participants performed the Montreal Battery of Evaluation of Amusia [45]; their individual scores for the full battery and for the contour test (as used by [16]) were below cut-off for the amusic group (20.68±1.60 and 18.55±2.16), but not for the control group (27.23±1.12 and 27.55±1.75). Written informed consent, as approved by the French ethics committee, Comité de Protection de Personnes Sud-Est II, was obtained from all participants.

**Table 4:** Number of participants per group, mean age (years), mean education (years), mean duration of musical training (years or level*) as well as mean scores obtained on the Montreal Battery of Evaluation of Amusia (MBEA), for the entire test (global score) or the subtest focusing on the processing of musical contour (as used by Douglas and Bilkey [16]).

		n	Gender	Age	Education	Musical training	MBEA**		
							Full battery	Contour test	
Experiment 1: Bisection tasks	Amusics	11	5M, 6F	34.73 (9.65)	15.00 (1.73)	0.77 (1.60)	20.68 (1.60)		18.55 (2.16)
	Controls	11	5M, 6F	35.00 (10.53)	14.18 (2.60)	0.36 (0.92)	27.23 (1.12)		27.55 (1.75)
Experiment 2: Mental rotation task	Amusics	9	4M, 5F	65.22 (3.80)	17.3 (2.78)	1.89* (1.05)	19.43 (2.17)		19.56 (2.83)
	Controls	9	2M, 7F	63.89 (4.96)	16.22 (2.17)	1.89* (1.05)	26.70 (1.09)		26.22 (2.68)

Note: Standard deviation is in parentheses.

*Musical training is classified into 5 levels: 1  =  less than one year, 2 =  1–3 years, 3 = 4–6 years, 4 = 7–10 years, and 5 = more than 10 years.

**The maximum score is 30 and cut-off scores below which an individual is considered amusic are 23 and 22, for the full battery and the contour subtest, respectively.

Amusics were impaired in pitch processing, as revealed by performance in a variant of a pitch change detection task (following the procedure of [4], and using a standard pitch at 215 Hz instead of 1047 Hz). The task consisted in detecting a pitch change in a sequence of five tones. In half the sequences, the fourth tone was changed by 25, 50, 100, 200, or 300 cents (100 cents correspond to one semitone). As expected, for small pitch changes (25 or 50 cents), amusics showed impaired performance (expressed here as proportion of Hits - False Alarms) with an average performance of 0.45, relative to controls who had an average performance of 0.67, *t*(20)  = 2.49, *p*<.02.

#### Material and Procedure

Participants were instructed to mark the midpoint of a straight 20-cm line (Figure 1A) or a 20-cm line made of letter strings spelling out the number words two and nine in French (Figure 1B). All lines were presented in black on white paper sheets of format A4, in horizontal orientation. Participants were instructed to mark the midpoint of the line with a pen, without considering words or letters. For each condition, fifteen lines were used. Participants first completed the task on straight lines only, then on the number lines. After all trials with the straight line, trials alternated between small and large number lines. Whether participants started with either small or large numbers was counterbalanced across participants.

### Experiment 2: Mental rotation task

#### Participants

The amusic group and the matched control group were different from those tested in Experiment 1 and consisted of 9 adults each, selected with the same constraints as described in Experiment 1 (see Table 4). Participants individual scores for the full battery and the contour test (as used by [15]) were below cut-off for the amusic group (19.43±2.17 and 19.56±2.83), but not for the control group (26.70±1.09 and 26.22±2.68). Written informed consent approved by the ethics committee of the University of Montreal was obtained from all participants. These amusics were also impaired in pitch processing, as revealed by their performance in the pitch change detection task of Hyde and Peretz [4]. The task consisted in detecting a pitch change in a sequence of 5 tones, as described in Experiment 1, but with a standard pitch at 1047 Hz. For small pitch changes (25 or 50 cents), the amusic group showed impaired performance (mean: 0.46 in proportion of Hits - False Alarms) in comparison to the controls of Hyde and Peretz [4] who performed at ceiling (0.95).

#### Material and Procedure

Two Shepard-Metzler cube figures were displayed on a computer screen side by side simultaneously on each trial (Figure 2). All forms were presented with the major axis vertically oriented. In half of the trials, the two forms had the same three-dimensional structure and the left and right forms were rotated in depth relative to each other by 0, 60, 120, or 180 degrees. In the other half of the trials, the two forms were mirror-images that could not be rotated into one another (and although we rotated the images in depth, as for same-structure pairs, there was no clear way to define an orientation difference for these trials). Participants were asked to determine if the two objects were identical or different as accurately as possible. They were familiarized with the task with 24 practice trials. Feedback was given throughout the experiment. The task was computerized with participants responding by key presses with timing approaching millisecond accuracy (instead of verbal responses timed with a stopwatch as in [16] on 160 trials (instead of 20 trials in [16]).
